# Feature-Based Fusion Using CNN for Lung and Heart Sound Classification †

**DOI:** 10.3390/s22041521

**Published:** 2022-02-16

**Authors:** Zeenat Tariq, Sayed Khushal Shah, Yugyung Lee

**Affiliations:** Department of Computer Science and Electrical Engineering, University of Missouri-Kansas City, Kansas City, MO 64110, USA; sayed.shah@unt.edu (S.K.S.); leeyu@umkc.edu (Y.L.)

**Keywords:** lung sound detection, heart sound detection, convolutional neural network, model fusion, multi-features

## Abstract

Lung or heart sound classification is challenging due to the complex nature of audio data, its dynamic properties of time, and frequency domains. It is also very difficult to detect lung or heart conditions with small amounts of data or unbalanced and high noise in data. Furthermore, the quality of data is a considerable pitfall for improving the performance of deep learning. In this paper, we propose a novel feature-based fusion network called FDC-FS for classifying heart and lung sounds. The FDC-FS framework aims to effectively transfer learning from three different deep neural network models built from audio datasets. The innovation of the proposed transfer learning relies on the transformation from audio data to image vectors and from three specific models to one fused model that would be more suitable for deep learning. We used two publicly available datasets for this study, i.e., lung sound data from ICHBI 2017 challenge and heart challenge data. We applied data augmentation techniques, such as noise distortion, pitch shift, and time stretching, dealing with some data issues in these datasets. Importantly, we extracted three unique features from the audio samples, i.e., Spectrogram, MFCC, and Chromagram. Finally, we built a fusion of three optimal convolutional neural network models by feeding the image feature vectors transformed from audio features. We confirmed the superiority of the proposed fusion model compared to the state-of-the-art works. The highest accuracy we achieved with FDC-FS is 99.1% with Spectrogram-based lung sound classification while 97% for Spectrogram and Chromagram based heart sound classification.

## 1. Introduction

Cardiovascular and respiratory disease are the top two global causes of death according to World Health Organization [1]. Furthermore, the Centers for Disease Control and Prevention reported heart disease is the leading cause of death (one in every four deaths) for adults in the United States. In particular, there is an increased risk of severe COVID-19 infection of that individual with certain medical conditions, such as heart diseases or chronic obstructive pulmonary disease (COPD) [2].

The expenses of health care have been rapidly increased in the United States [3]. Due to the rapid surge of medical care costs, many people cannot afford health care and may have proper medical treatment. A physician can diagnose using a standard clinical stethoscope by hearing sounds from the human body. An auscultatory method has been applied widely by physicians to examine lung sounds associated with different respiratory symptoms. The auscultatory process has been the easiest way to diagnose patients with respiratory diseases, such as pneumonia, asthma, and bronchiectasis [4]. However, the sound quality is quite a noise or too weak to hear, sometimes due to the complexity of the sound patterns and characteristics. Thus, the manual process takes much time and effort for a physician to detect the condition using a stethoscope accurately [5]. For example, wheezing sounds could not accurately be identified in a series of the pulmonary disease sounds [6].

Similarly, the physicians use cardiac auscultation to evaluate cardiac functions and detect diseases [7]. However, it is a difficult task to manage this method manually at present. A signal produced by these heart sounds is recorded and is known as Phonocardiography (PCG). These signals are highly potent for detecting various heart diseases and are not costly, unlike the electrocardiogram (ECG) signals identified through machines. However, it takes much time to analyze the signals. Hence, deep learning analytic may play an essential role in interpreting the sound signals where corrective measures can be made for physicians’ diseases.

There have been significant recent advances in deep learning and the potential of the deep learning model for various medical applications. Recently, there has been increasing attention for the classification of human body sounds for clinical conditions in the medical domain [8,9,10]. Advanced technologies are essential to achieving the improvement of lifestyle and health care. Some of these applications are ambient assisted living systems [11,12], fall detection [13], voice disorders [14], and heart condition detection [15]. These systems are useful in the early detection of different types of disease through human body sounds, which ultimately improves healthcare. More specifically, an extensive investigation in a partnership among researchers, health care providers, and patients is integral to bringing precise and customized treatment strategies in taking care of various diseases.

Recently, an electronic stethoscope, similar to the design of standard clinical stethoscopes, was designed to enhance the quality of body sounds through filtering or amplification and then extract features from the sounds for the automatic diagnosis of heart or lung conditions using deep learning algorithms. If a deep learning-based diagnosis of heart or lung disease can increase precision and productivity, we can reduce healthcare costs and improve healthcare quality. Moreover, deep learning is a branch derived from machine learning. It allows the computational models, which consist of several layers of processing used to learn the data representations over multiple levels of abstractions. It has attracted a lot of attention due to its high performance in classification. These learning techniques are among the fastest-growing fields at present in the area of audio classification [16]. Some studies reported that the deep learning models outperform humans due to the ability to filter the noise and intensive learning ability [17,18].

The human body sounds for classification are too complicated to understand the hidden patterns of data. The image-based sound classification was introduced to effectively captures the diverse patterns in the dataset [19,20,21]. As image-based sound classification was introduced, more diverse fusion approaches were also introduced to improve the performance of classification. These methods include the modality-based fusion [22], feature level fusion [23], network-level fusion [24,25] and methodology-based fusion [26]. Among various fusion techniques for sound classification, the most popular are those based on features and multi-modality. However, not much work has been conducted on the general fusion approach, such as network-level fusion using multi-features, which can apply to more diverse datasets or applications.

**Figure 1:**
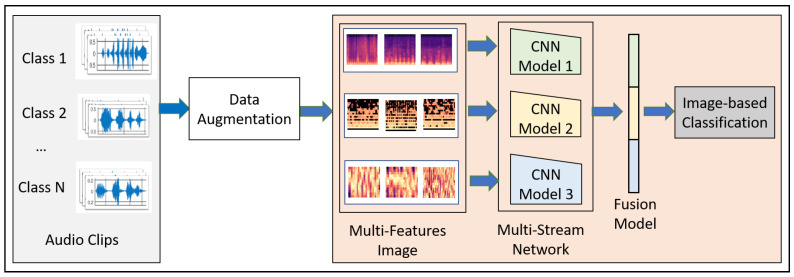
Fusion-Based Disease Classification Architecture.

**Figure 2:**
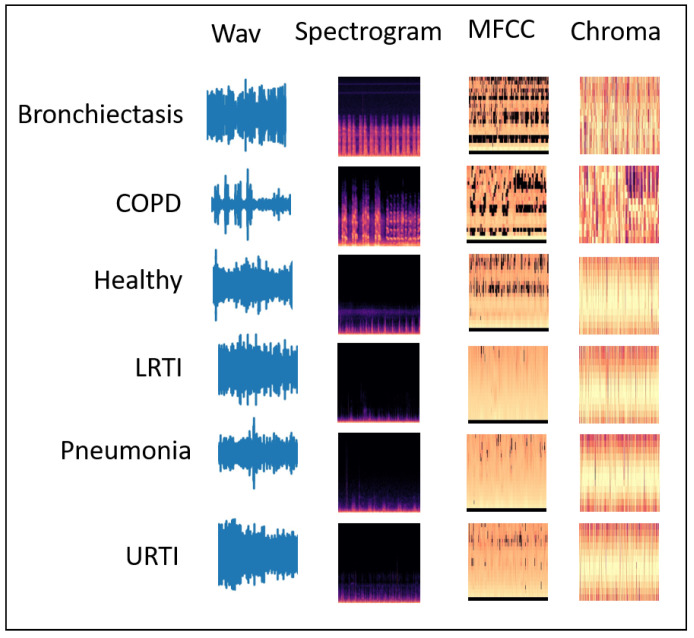
Lung Sound Features: (1) Wav (2) Spectrogram (3) MFCC (4) Chromagram.

**Figure 3:**
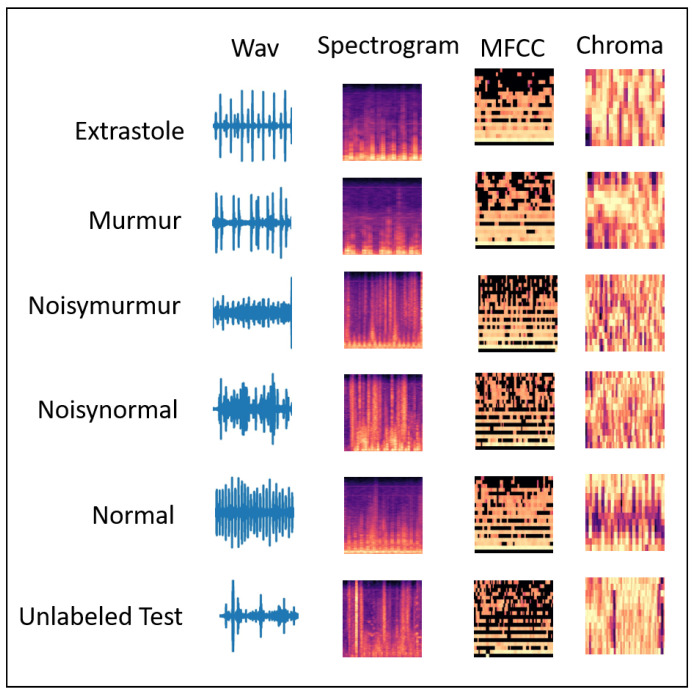
Heart Sound Features: (1) Wav (2) Spectrogram (3) MFCC (4) Chromagram.

This paper proposes a novel convolutional neural network model-based fusion called “Fusion-based Disease Classification (FDC)”. The model architecture is shown in Figure 1. Our contribution to this paper can be summarized as follows:

- We designed a feature-based fusion model (FDC-FS) transferred from the three feature-based convolutional neural network models to classify lung and heart disease.
- We found that it is more effective to classify heart or lung diseases with images transformed from three different sound features, i.e., Spectrogram, MFCC, and Chromagram (shown in Figure 2 and Figure 3).
- The three types of data augmentation, such as Noise, Pitch-Shift, and Time-Stretch, have been effectively applied to the audio dataset for optimal deep learning training and testing performance.
- A comprehensive experimental results with the lung and heart sound datasets confirmed the superiority of the proposed FDC model over the state-of-the-art methods.

The remainder of the paper is organized as follows: Section 2 describes the related work on different deep learning models and lung and heart disease classification techniques. Section 4 discusses the methodology of the proposed model, FDC-FS. Section 4 describes the results and evaluation of the classification model. Section 5 discusses the state-of-the-art comparison for both lung and heart datasets. Finally, Section 6 presents the conclusion and future work.

## 2. Related Work

### 2.1. Lung Disease Classification

Rocha et al. [27] developed classification models for the diagnosis of chest conditions using a stethoscope for the environmental sounds. First, they created a database of lung sounds, which consisted of 920 samples for different categories (i.e., COPD, Healthy, etc.). The second task of the challenge was to extract the features and classify the sounds according to the nature of sound (Wheezes, Crackles, or both). Finally, they conducted feasibility studies on machine learning algorithms, such as support vector machine (SVM) and artificial neural networks (ANN) using features, such as MFCC, spectral features, energy, entropy, and wavelet coefficients. However, they have not overcome the data issues. Unlike this study, we extracted multiple image features from the same datasets, improved the data issues using data augmentation techniques, and obtained better results.

Several data augmentation techniques were applied to classify lung diseases using sounds [28,29,30,31]. Dalal et al. [32] explored four machine learning approaches for lung sound classification using lung dataset [33]. This study used data augmentation and extracted Spectrogram, MFCC, and LBP features using multiple machine learning algorithms, such as Support Vector Machine (SVM), K-Nearest Neighbor (KNN), Gaussian Mixture Model (GMM), and Convolutional Neural Network (CNN). Among these models, CNN outperformed all other classifiers with an accuracy of approximately 97%. However, their machine use was very high, applying almost 1 million or more epochs. On the contrary, we achieved higher accuracy than the study, with very low machine use and only 100 epochs with low parameter consumption.

The review in [34] mentioned several feature extraction and classification techniques for obstructive pulmonary diseases such as COPD and asthma. The process involves several traditional and deep learning classification techniques, such as K-Nearest Neighbor (KNN), Artificial Neural Network (ANN), Deep Neural Network (DNN), and Convolutional Neural Network (CNN) and feature extraction through signals such as Fast Fourier Transform (FFT), Short Time Fourier Transform (STFT), Spectrogram, and wavelet transform. For example, the best accuracy for CNN was approximately 95%.

Hai et al. [35] proposed a novel solution for lung sound classification using a publicly available dataset. The dataset was divided into three categories such as wheezes, crackles, and normal sounds. They proposed a detection method using optimized S-transformed (OST) and deep residual networks (ResNets). They performed preprocessing on the audio samples using OST for rescaling the features for ResNets and used a visual-based approach. Their experimental results showed the best multi-classification model with accuracy (98.7%).

Fateh et al. [36] proposed a pre-trained CNN model to extract deep features using the ICBHI challenge dataset. The dataset consists of crackles, wheezes, normal, and wheezes plus crackles categories. First, they used the visual approach with spectrogram images generated from lung sounds by extracting the features. Then, they used the deep features as the input of the Linear Discriminant Analysis (LDA) classifier using the Random Subspace Ensembles (RSE) method. As a result, their model improved the classification accuracy from 5% compared to the existing methods.

Demir et al. [37] proposed two approaches using CNN for the classification of lung diseases using the ICBHI lung sound dataset. The dataset consists of 4 classes with 6898 recordings. First, they converted the lung sounds to spectrogram images using the Short Time Fourier transform (STFT) method. Their first approach is classifying with the SVM based on the features extracted from a pre-trained CNN model. Then, the pre-trained CNN model was fined tuned using the transfer learning method for spectrogram images. The best accuracy for the proposed first and second methods are 65.5% and 63.09%, respectively.

Samiul et al. [38] proposed a lightweight CNN model for detecting respiratory diseases through lung sounds. They designed a hybrid scalogram-based approach using the ICBHI 2017 lung sound dataset by using the empirical mode decomposition (EMD) and the continuous wavelet transform (CWT). As a result, the three-class chronic and the six-class pathological classification accuracy were 98.2% and 98.72%, respectively, with 3M trainable parameters. However, we achieved a better accuracy with lower trainable parameters.

Elmar et al. [39] presented an approach for multi-channel lung sound classification using spectral, temporal, and spatial information. They proposed a convolutional recurrent neural network (CRNN) using spectrogram features to classify lung sounds collected from 16 channel recording devices. Their CRNN model obtained an F1-score of 92% for the binary classification.

Luay et al. [40] proposed homogeneous ensemble learning methods to perform multi-class classification of respiratory diseases. They also used the ICBHI challenge dataset, including 1176 recordings and 308 clinically obtained lung sounds. They used entropy features for machine learning models such as SVM, KNN, and Decision Tree. Among these three models, SVM received the best average accuracy of 98.20%.

### 2.2. Heart Disease Classification

Potes et al. [41] proposed a feature-based ensemble technique for the classification of normal vs. abnormal heart sounds. First, they extracted 124 time-frequency features such as MFCC from the phonocardiogram (PCG) signals. Then, they used the combination of AdaBoost classifier and CNN classifier to classify the heart sounds. The overall accuracy achieved was 86%. Zhang et al. [42] proposed a method for heart sound classification using a convolutional neural network (CNN). The spectrogram features were extracted from the cycles of sound signals for different positions of pre-trained CNN, and the classification model was based on a support vector machine (SVM). They reported the precision of 77% and 71% for the two datasets with 4 and 3 classes, respectively.

Bozkurt et al. [43] focused on segmentation and time-frequency components for the CNN-based designs. The Mel-spectrogram and MFCC features were extracted from heart sound data using the PhysioNet dataset [44]. They also performed the data augmentation by changing the sampling rate with a random value in range. The overall accuracy achieved was 81.50%. Shu et al. [45] proposed a novel deep WaveNet model for the classification of heart sounds. They used the dataset, composed of five categories with the 1000 PCG recordings, and obtained the overall highest training accuracy of 97%. Muqing et al. [46] proposed a combined model with a convolutional neural network (CNN) and recurrent neural network (RNN) based on the MFCC features for heart sound classification. Their results for the heart sound classification with pathological or non-pathological categories showed the accuracy of 98% with the PhysioNet database.

The authors [47] evaluated the challenges and future directions in the application of supervised learning to cardiovascular beats analysis in order to provide a critical guidance for future research. Despite substantial advancements in this domain, there are still constraints due to a lack of available datasets, inadequate learning, and a paucity of effective algorithms. Our study’s major objective is to increase the validity of heart sound recognition. The present investigation included an in-depth analysis and evaluation of recent deep learning methods, with a concentration on the convolutional neural network (CNN) and recurrent neural network (RNN) techniques that have evolved progressively over the last several years.

Acharya et al. [48] proposed Convolutional Neural Network having nine layers for the classification of heart heartbeat signals, such as non-ectopic, supraventricular ectopic, ventricular ectopic, fusion, and unknown beats. Oh et al. [49] developed hybrid models, i.e., multiple layers of CNN and max-pooling and LSTM as the end layer, to extract the temporal information from the features from the ECG dataset and classify arrhythmia from ECG segments. Rajpurkar et al. [50] developed a 34-layer CNN model for the diagnosis of heart diseases such as arrhythmia with the ECG data, recorded with a single lead heart monitor. However, some issues arise in deep learning modeling with the data, including the limited amount of data for heart conditions, low quality with noise, and significant data variations. We also faced similar problems and addressed them in our work.

## 3. Methodology

We discuss the overall design goals for the FDC network (shown in Figure 1). The modeling process of FDC includes five stages. First, we apply data augmentation techniques onto the audio data to handle the data issues and improve heart and lung condition detection accuracy. Second, we extract the three types of unique and dominant features inherent from the audio data, i.e., Spectrogram, Mel-frequency cepstral coefficient (MFCC), and Chromagram. Third, we convert the extracted features in the form of the images and generate the feature vectors of the audio images in a color format. Fourth, we feed the image feature vector into the especially designed three convolutional neural network models (FDA-1, FDA-2, FDA-3). Finally, The fusion network model (FDA-FS) fuses these three models to optimize the learning performance for the heart and lung sound datasets.

### 3.1. Rationale of Design

The rationales of the FDC framework design are as follows: First, it is to enable the effective selection of features from the heart and lung sound data, which are highly noise and unbalanced. Several discrepancies existed in the datasets’ sources. We did not, however, cut or change the audio input; rather, we extracted the most salient qualities or patterns from the complete dataset. Second, it can transform the audio features into consistent and reliable forms, i.e., audio images. FDC supports a multi-modality capability of audio and image in feature extraction, modeling, and inferencing. Third, it is to design the three different network models to effectively learn unique and dominant features. Finally, it is to transfer learning by fusing the three network models into one model to improve the learning performance in lung and heart condition detection.

The differences between the samples of the signals (audio) and images of the same signals are significant, although they are from the same sources. Thus, different modeling techniques are needed to support the multi-modality. For example, if one is talking in a visual context, it means that sound is transparent while the image is considered as non-transparent (opaque) [51]. The pixel formation shows that whatever is the position of an object belongs to the same category. However, audio cannot quickly identify where it belongs due to its observed frequency in spectrogram features. The audio spectrogram depends on the magnitude of the frequency. Therefore, it is not easy to process the object or its combination in a sequence manner, and it is challenging to identify the simultaneous sounds represented in the spectrogram features.

When a neural network uses images for classification, it considers the sharing weights available from the images in two dimensions, i.e., the X and Y-axis. Thus, the image will carry the same information if it is stretched or repositioned regardless of the position and presence. However, in audio, the two-dimensional data represents the frequency and time. Therefore, if the audio is moved horizontally or vertically, the meaning of the audio can changes. Furthermore, even the spatial features of the sound can change if we increase or decrease the pitch. Therefore, the intentionally introduced invariance can change the meaning, and the neural network will not perform well as it should perform on the trained and augmented data.

In the image format, the extracted pixels can assume that the image belongs to the same class, while in audio, this is not true. Audio is composed of periodic sounds that are composed of frequency and harmonics. The harmonics are spaced apart from each other according to their nature. The combination of these harmonics usually determines the sound’s timbre. Suppose we assume from the physical point of view. In that case, the audio played to the audience will examine the type of sound only, while images typically contain a lot of parallel static information. The image classification depends on image features, such as brightness and resolution, among other features.

The design of the fusion-based FDC framework can be justified in terms of the classification effectiveness in terms of three perspectives: (1) Extracting features from the complex audio data of the time and frequent by transformation from audio to images. (2) Designing three specific deep neural networks to optimize their learning performance depending on their unique and dominant features. (3) Optimizing the learning performance through a fusion model by combining the three different models.

### 3.2. Data Augmentation

Deep learning relies on a large amount of data for more accurate classification. Therefore, we used several augmentation techniques for better classification, out of which we selected the best three methods, including background noise, time stretching, and pitch shifting. We designed these techniques to address the problems in the lung and heart audio classification.

We examined a range of alternative data augmentation techniques. Using a heuristic approach to produce the ideal output, we investigated and validated the augmentation. The experiments attempted to multiply the original file’s size tenfold and determined the highest degree of performance. We required a consistent level of research across all models and data, which is why the initial data set was tenfold augmented.

#### 3.2.1. Noise Distortion

We considered adding random noise to the audio samples to avoid overfitting during training. In this type, noise clips were randomly sampled [52] to be linearly mixed with the input signal represented as $y^{\prime }$. α is used to describe random weights along with specific factors that are denoted by *U* as shown in Equation (1).

(1) $$\begin{array}{c} Random-Weights:\alpha {∼}_{U}\left[0.001,0.005\right]\\ Input-Signal:y^{\prime }\leftarrow \left(1-\alpha \right)\cdot y+\alpha \cdot y_{\mathrm{noise}\;} \end{array}$$

#### 3.2.2. Time Stretching

Scaling the audio data horizontally by some stretching factor such as a_st>0 helps in increasing the size of the data for efficient classification. We applied time stretch (st) on the audio samples, which were later converted to images. It is to check if the meaning of the data remains the same as image data does not lose the information but changing the position of audio or slowing down the position as we generate the Spectrograms. We considered four different types of time stretch factor $n\in \left\{0.5,0.7,1.2,1.5\right\}$.

#### 3.2.3. Pitch Shifting

In this data augmentation technique, the audio samples’ pitch is either decreased or increased by four values (semitones) [53]. We assume that with the pitch shifting factor $a_{s}$, the artificial training data generated is Naug times larger than the original lung or heart sound data. The duration of audio samples is kept constant, like the actual audio samples, i.e., 10–90 s. For our experimentation, the value changed in semitones were in the interval $\left[-a_{s},a_{s}\right]$ for each signal. Factors of pitch shift are n∈{−3.5,−2.5,2.5,3.5}semitones.

### 3.3. Feature Extraction

We used the two datasets, i.e., lung and heart sound (for six categories for each in the lung dataset and the heart dataset). These datasets consist of sound clips that vary from 10 s to 90 s. However, to incorporate the consistent data in making a more accurate prediction model, we used the sliding window technique to make each clip of 3 s. The window size (i.e., 3 s) for optimal learning was chosen using a heuristic based on the distribution of signals from a small subset of the PCG input. Utilizing the spectrogram, MFCC, and chromagram approaches, we retrieved audio characteristics from the given input. Metadata describing the location of heart sounds or signal synchronization, as well as other insights, enabled the effective extraction of the different properties gained by these extraction strategies on the three-second PCG segments. The feature vectors of the extracted features for these three types are converted as JPG images in the dimension of [128 × 128] using the CV2 image and NumPy libraries.

#### 3.3.1. Spectrogram Generation

We used the spectrogram to generate a visual representation of a signal in the time-frequency domain. These are generated by the application of the short-time Fourier transform (STFT) [54]. According to the theorem, a single Fourier analysis may not see a nonstationary signal’s spectrum variation. The Fourier transform can be used to determine the frequency and sequence of signals and their changes over time. Hence, the spectrogram considers the stationary signal by computing the Fourier transform of the segmented signal into slices. The spectrogram can be calculated as:

(2) $${\mathrm{STFT}}_{x}^{f}\left(t,f\right)=\int_{\infty }^{\infty }[x\left(t\right)w\left(t-\tau \right)e^{-j2\pi ft}dt$$

where *x*(*t*) is the time-domain signal, τ is the time localization of STFT, and w(t−τ) is a window function to cut and filter the signal. The length of the window function must be selected and adjusted according to the signal’s length because it affects the time and frequency resolution [55]. The window size was determined using a heuristic based on the distribution of signals from a small subset of the input. To do this, we considered all possible window sizes and other criteria for selecting audio features in STFT. It should be noted, however, that the input data included just three seconds of each PCG signal. As a result, the maximum permissible window size was 3. The spectrogram was converted into a grayscale image, and the image will be used to generate a feature vector that will be feed for deep learning.

The scaling process is applied to the spectrogram to expand the values range between 0 and 255 because the range of the spectrogram is usually comprehensive. The method of scaling is done in a linear manner, which can be expressed as follows:

(3) $$S\left(m,n\right)=\frac{\left|Spec(m,n)\right|}{max|Spec|}\times 255$$

where *Spec*(*m*, *n*) is the value of the spectrogram and *S*(*m*, *n*) is the expanded value from a spectrogram.

#### 3.3.2. Mel-Frequency Cepstral Coefficient

The Mel-frequency Cepstral Coefficient (MFCC) coefficients are a set of discrete cosine transform (DCT) derived from a type of cepstral representation of the audio clip. The frequency warping allows a better representation of sound by containing the difference between the cepstrum and the Mel-frequency cepstrum. It computed through logarithmic spectrum scale after it was transformed to the Mel scale [56] calculated as:

(4) $$\mathrm{mel}\left(f\right)=2595\log_{10}\left(1+\frac{f}{700}\right)$$

For the input signal y(n), an N-point discrete Fourier transformation (DFT) is given as:

(5) $$Y\left(k\right)=\sum_{n=1}^{M}y\left(n\right)\cdot e^{\left(\frac{-j2\pi nk}{M}\right)}$$

MFCCs are commonly derived as follows: first, we obtain the spectrum by taking the Fourier to transform to a signal. Second, we map the spectrum onto the Mel scale and then take a log-based transform of the Mel-frequency scaled spectrums. Finally, we take the discrete cosine transform of the Mel-log-frequency scaled spectrums and amplitude the spectrum.

#### 3.3.3. Chromagram

Chromagram features or Chromagram are Pitch Class Profile whose pitches can be meaningfully categorized. The Chromagram technology was applied to generate a robust set of acoustic features by capturing harmonic and melodic characteristics of music in a signal whose pitches can be classified in the categories of lung or heart sounds. Since the heart and lung sounds have subtle differences in pitch, Chromagram has features that make it a good source of lung and heart sound classification.

### 3.4. Classification Model

#### 3.4.1. Overview of the FDC Model

Convolutional neural network (CNN) has been recognized as a popular and powerful deep neural network model in audio and image classification applications. We developed a fusion model based on CNN-based architecture for heart and lung disease classification. Our model is a 2D CNN model composed of the input layer, convolutional 2D layer, max-pooling layer, and fully connected layers. The invention is a fusion model, FDC-FS, by combining multi-featured models, including FDC-1, FDC-2, and FDC-3. The fusion was conducted on their final layer to append all model parameters. The three models FDC-1, FDC-2, FDC-3, were trained concurrently and independently. After completing the single model development process, we combined the models to generate the FDC-FS fusion model. The general hyper-parameters are shown in Table 1. The detailed description of each model in given in Section 3.4.2, Section 3.4.3, Section 3.4.4 and Section 3.4.5. These four models’ (FDC-1, FDC-2, FDC-3, FDC-FS) accuracy was summarized in Section 4.

There are two essential components in the design of multi-feature models with CNNs. (1) the feature extractor collected features (Spectrogram, MFCC, and Chromagram) from the audio signals and transformed the features into images to generate visual feature vectors. (2) Each model (FDC-1, FDC-2, FDC-3) was uniquely designed to optimize learning for the specific features (Spectrogram, MFCC, and Chromagram), respectively, which is composed of multiple convolutional and pooling layers, activation, and fully connected layers with several hidden units. Finally, the fusion model was built by composing the features from these three models. After the models were trained, the visual feature vectors of the input signals were classified by the models into their appropriate categories. 

The mathematical form of the convolutional layers is given in Equations (6) and (7)

(6) $$\left[x_{i,j,k}^{l}=\sum_{a}\sum_{b}\sum_{c}w_{i,j,k}^{\left(l-1,f\right)}y_{i+a,j+b,k+c}^{\left(l-1\right)}+{bias}^{f}\right]$$

(7) $$\left[y_{i,j,k}^{l}=\sigma \left(x_{i,j,k}^{\left(l\right)}\right)\right]$$

The output layer is represented by $y_{i,j,k}^{l}$ where as the 3-dimensional input tensor is denoted by i,j,k. The weights for the filters are denoted by $w_{i,j,k}^{\left(l\right)}$ and $\sigma (x_{i,j,k}^{\left(l\right)})$ describes the sigmoid function for linear activation. The fully connected is the final layer represented by Equations (8) and (9).

(8) $$\left[x_{i}^{\left(l\right)}\sum_{j}w_{i,j}^{l-1}y_{j}^{l-1}+{bias}_{j}^{l-1}\right]$$

(9) $$\left[y_{i,j,k}^{l}=\sigma \left(x_{i,j,k}^{l}\right)\right]$$

Our fusion model FDC-FS is composed of three different models, such as FDC-1, FDC-2, and FDC-3. They consist of the convolutional layers enclosed by the max pool layer, followed by fully connected layers, including dropout, batch Normalization, rectified linear units (ReLU), and LeakyReLU. During the extraction of features, we used the window size and hop size of 23 ms. As the sound clips vary between 3 and 5 s, that is why we kept the extraction to 3 s to make every bit of the sound clip usable. In addition, we reshaped the input taken from the sound clips to $X\in R^{128\times 128}$ shape. Further, we sent these reshaped features to the classifier to predict heart or lung diseases.

#### 3.4.2. FDC-1 Model

The FDC-1 model is designed for the classification based on the image feature vector of the three audio features (Spectrogram, MFCC, and Chromagram) for the given datasets, using convolutional neural network architecture with a total of five layers. Among the five layers, 3 are convolutional layers, and 2 are dense layers. We considered rectified linear units (ReLU) as the activation function between layers, a max-pooling is also applied, and we also used dropout in different layers to avoid overfitting. The total number of trainable parameters based on the five layers of architecture is 241,174 (0.24 M). The hyper-parameters are shown in Table 2.

The first layers of the FDC-1 model consist of 24 filters with a 5 × 5 receptive field. The layer is also followed by a (4 × 2) strided max-pooling function. The activation function used in this layer is rectified linear units (ReLU). The second layer of FDC-1 is composed of 48 filters with 5 × 5 receptive files. It is followed by 4 × 2 strided Max Pooling and ReLU activation. The padding for these layers is kept as “valid”. The third layer of FDC-1 consists of 48 filters with 5 × 5 receptive fields. The layers have “valid” padding, which is followed by the ReLU activation function. After the activation, the output is flattened, and the dropout of factor “0.5” is applied to avoid overfitting the output from layer to layer. The fourth layer is the first dense layer which is also called the hidden layer. It consists of 64 hidden units followed by ReLU activation and dropout rate of 0.5 to avoid overfitting the output result to the next layer. The fifth layer is a final dense layer that consists of output units. The output units are always equal to the number of classes used in the dataset. The last layer is followed by the “Softmax” activation function.

#### 3.4.3. FDC-2 Model

The FDC-2 model is designed for classification based on the image feature vector of the three audio features (Spectrogram, MFCC, and Chromagram) for the given datasets. It is based on a convolutional neural network architecture consisting of 4 layers, including two convolutional layers, two hidden layers, and an L2 regularizer on the first layers to reduce likelihood and bias among the inputs. In addition, this model consists of Max Pooling to reduce unwanted features for training, dropout to avoid overfitting, ReLU, and Softmax activation. Feature vector flattening is also considered to convert 2-dimensional features to 1-dimensional features. The total number of trainable parameters based on the four layers of architecture is 879,430 (0.87 M). The overall hyper-parameters are shown in Table 3.

In the first layers of FDC-2, the layers take 32 filters with 3 × 3 receptive files. The first layers also consist of the L2 regularizer norm with the value of “0.0005”. Then, Strided Max Pooling of 4 × 2 follows it. Finally, ReLU is used as an activation function. FDC-2 takes 48 filters with 3 × 3 receptive filed and “valid” padding in the second layer. It is pursued by the ReLU activation function and Max Pooling of 4 × 2. After all the operations above, the 2-Dimensional input is flattened to 1-Dimensional and passed on to the hidden layers, i.e., dense layers. A dropout follows the flatten with a rate of 0.5 to avoid overfitting the input. The third layer is the first hidden (dense) layer of FDC-2 with hidden units of 64, followed by ReLU activation and dropout with a rate of 0.5. The fourth layer is a dense layer consisting of the output units, which is equal to the number of classes available in the dataset. The final activation function is Softmax.

#### 3.4.4. FDC-3 Model

The FDC-3 model is designed for the classification based on the image feature vector of the three audio features (Spectrogram, MFCC, and Chromagram) for the given datasets, focused more on in-depth training and eventually reducing the number of trainable parameters. This model is composed of 8 convolutional layers and one dense layer. The layers consist of padding, ReLU, softmax activation, Max Pooling, Global Average Pooling, Batch Normalization, and dropout. The Batch Normalization is used to train the model intensely, and in return, it standardizes the input in a layer for each mini-batch. Hence, it has a perfect effect on the learning process, reducing the number of trainable parameters. The total number of trainable parameters based on the nine-layer architecture given below is 362,214 (0.36 M). FDC-3 hyper-parameters are shown in Table 4 in details.

The first and second layers of FDC-3 have 32 filters with 3 × 3 receptive fields and some padding and ReLU as activation function. Both layers also consist of 2 × 2 strided Max Pooling. Batch Normalization follows both layers to perform deep training and reduce the trainable parameters. However, the second layer is following by a dropout of 0.25 to overfitting the input to the next layer. The third-to-sixth layers of FDC-3 are the same as the first and second layers, but the third-to-sixth layers take 64 filters with 3 × 3 receptive fields. They use the same strided max-pooling, padding, activation, dropout, and Batch Normalization for deep training. The seventh and eighth layers of FDC-3 take 128 filters with 3 × 3 receptive files, followed by the same padding and ReLU activation function. Batch Normalization follows the activation function in both layers. However, the eighth layer is followed by the dropout of rate 0.25. Finally, the last convolutional layer is also followed by Global Average Pooling before the input is ready for output classification. The ninth layer is the final and only the dense layer in this architecture, consisting of output units equal to the number of classes in the dataset.

#### 3.4.5. FDC-FS Model

**Figure 4:**
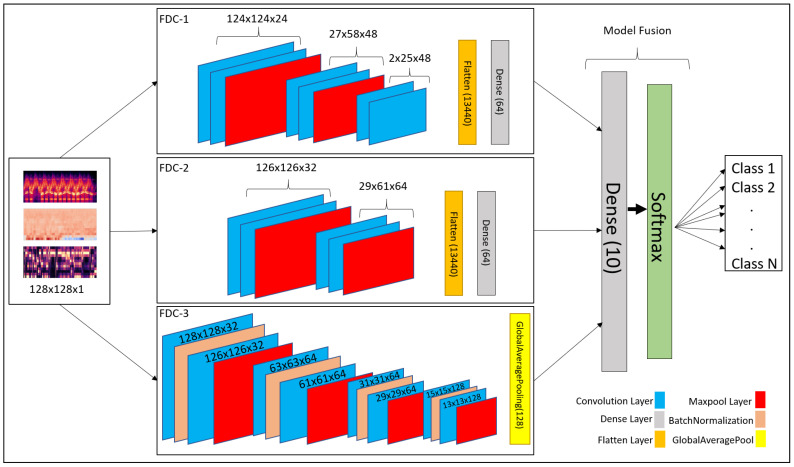
Overall FDC-FS Architecture Composed of FDC-1, FDC-2, and FDC-3.

The FDC-FS model is a fusion model resulting from transfer learning from all three models (FDC-1, FDC-2, FDC-3). Its architecture is composed of the softmax activation and dense layers consisting of the output units equal to the number of classes in the dataset at the last layer. Therefore, FDC-FS is composed of 13 convolutional layers and 3 dense layers model, more specifically three convolutional layers from FDC-1, two convolutional layers from FDC-2, eight convolutional layers from FDC-3, one dense layer from FDC-1, and one dense layer from FDC-2, and a final dense layer of an output unit. The FDC-FS’s total trainable parameters for six classes are 1,482,806 (1.4 M). Table 5 shows the hyper-parameters of the final convolutional architecture, which is a fusion of our novel three architectures shown in Figure 4.

## 4. Result and Evaluation

We conducted comprehensive experiments for the FDC models using the lung and heart sound datasets [27,57]. We now present the results obtained from the experiments with the three FDC models and the fusion model (FDC-FS) using the original and augmented datasets of the lung and heart datasets. The results includes the accuracy, loss, and class-wise accuracy for original and augmented datasets. The experimental results have been obtained as compared to the state-of-the-art methods.

### 4.1. Experimental Setup

We conducted most of the experimentations using Google research collaboratory with 12 GB NVIDIA Tesla K80. For the data augmentation and feature extraction, we used the NVIDIA GeForce ^®^ GTX 1080 Ti, packed with 11 Gbps GDDR5X memory and an 11 GB frame buffer. The number of epochs was set at 50 while avoiding overfitting, and a batch size of 64 was considered. However, for the training for the augmented datasets, the epochs were set at 30 with a 128 batch.

The model training was set to 80% training and 20% testing. From the 80% training data, we further split it into 80% training and 20% validation (64% training, 16% validation, 20% testing). This is to note here that testing data are not a subset of training data. After the data were split, the testing data was never seen by the previous model. We reported classification accuracies for training and testing. The class-wise accuracy was also reported for four different models (FDA-1, 2, 3 & FS), two types of data (original and augmented), and for two different datasets (lung and heart sound). We observed that FDC-FS performed the best compared to others.

### 4.2. Dataset

*Heart Sound Dataset:* The heart dataset consists of 656 audio recordings for different heart classes such as *Extrastole*, *Murmur*, *Noisy Murmur*, *Noisy Normal*, *Normal*, *Unlabeled test*. The author of the dataset [57] made this dataset public for two challenges, using an iPhone app and a digital stethoscope. The initial dataset has both clean and noisy data without any data synthesis. To increase the accuracy of the data, data augmentation techniques were added to the initial heard sound samples. After applying the data augmentation to the initial data, the total number of files increased to 7216 as shown in Table 6.

*Lung Sound Dataset:* The research team from Greece and Portugal created the lung dataset [27]. There are 920 annotated recordings, ranging from 10 s to 90 s (the total of 5.5 h), obtained from 126 patients using a digital stethoscope. Unfortunately, the complete dataset was not released. Therefore, the publicly available dataset used for lung sound classification modeling is mainly limited in data amount and sound quality. To overcome the data issues, we applied two approaches to balance the dataset: (1) The “Synthetic Minority Oversampling Technique (SMOTE)” replicates the same sample several times to balance the dataset with other classes. Most of the state-of-the-art research use SMOTE in their works. The second approach is to consider weighted average as our testing accuracy for the models. We further applied data augmentation techniques to generate synthesized data. Table 7 shows the amount of the original and augmented data. We removed the *Asthma* category having only a single recording from the dataset. The number of the original recordings was 919 for six categories (*Bronchiectasis*, *COPD*, *Health*, *LRTI*, *Pneumonia*, *URTI*) while the number of augmented recordings are 10,109.

**Figure 5:**
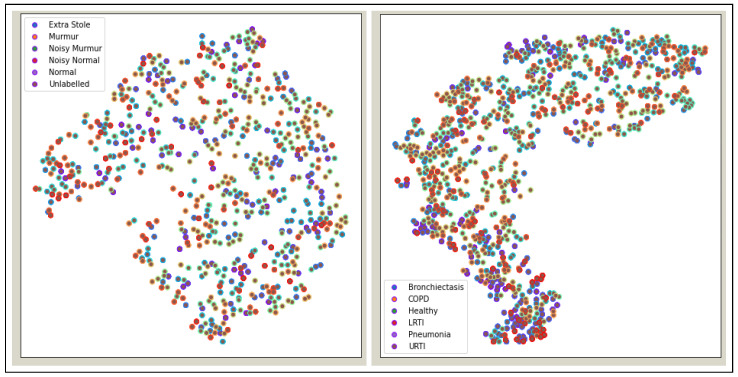
t-SNE Visualization: Heart Sound Dataset and Lung Sound Dataset.

Figure 5 shows the t-Distributed Stochastic Neighbor Embedding (t-SNE) visualization for heart and lung sound dataset. It can be seen that the Lung dataset is very dispersed, and classes are mixed up with each other.

### 4.3. Classification Results and Evaluations

**Figure 6:**
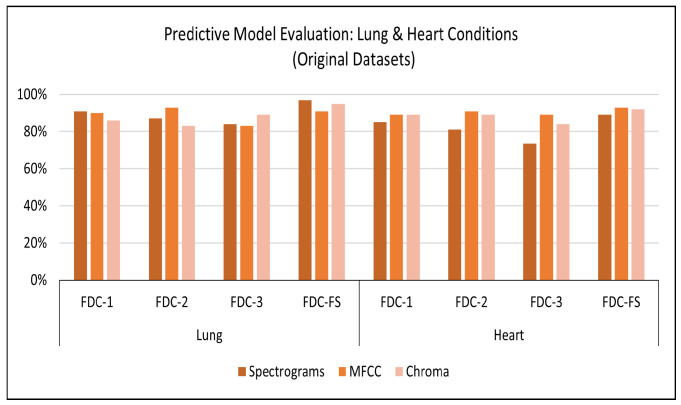
Accuracy for Lung/Heart Condition Detection (Original Dataset).

**Results on Original Lung Dataset:** Based on the setup explained above, we obtained the accuracy performance for the four models (FDC-1, FDC-2, FDC-3, and FDC-FS). FDC-FS model obtains the highest accuracy model in all three feature cases. Specifically, the highest accuracy is achieved by Spectrogram 97%, while MFCC reported accuracy of 91% and Chromagram reported accuracy of 95%. Table 8 offers the accuracy of the lung original dataset. The classification results for the original lung dataset are shown in Figure 6. Class-wise accuracy for all models based on the original lung dataset is shown further on in this paper.

**Results on Original Heart Dataset:** Our experimental results are based on 50 epochs and 64 batch sizes and the categorical cross-entropy for the data validation. The average times taken for training the model were from 7 s to 1 min for the FDC models. Table 8 offers the accuracy of the heart original dataset. The classification results for the original heart dataset are shown in Figure 6. During the training and testing, we observed that FDC-FS performed the best compared to all others; specifically, we obtained the highest accuracy of 93% for MFCC. For the individual feature-model evaluation, the highest accuracy of Spectrogram was 85% with FDC-1; for MFCC, it was 91% with FDC-2. Chromagram was the accuracy of 89% with FDC-1 and FDC-2.

**Figure 7:**
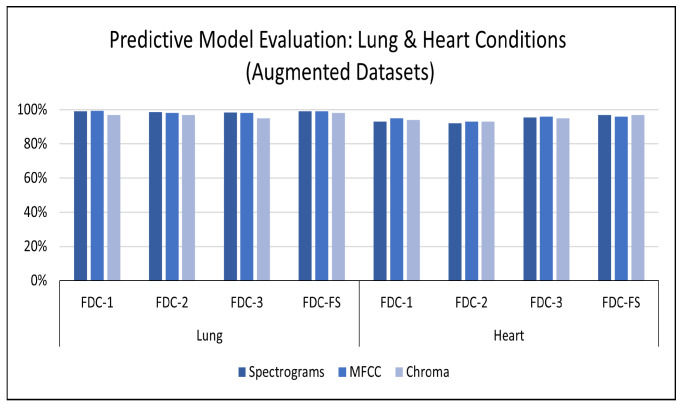
Accuracy for Lung/Heart Condition Detection (Augmented Dataset).

**Figure 8:**
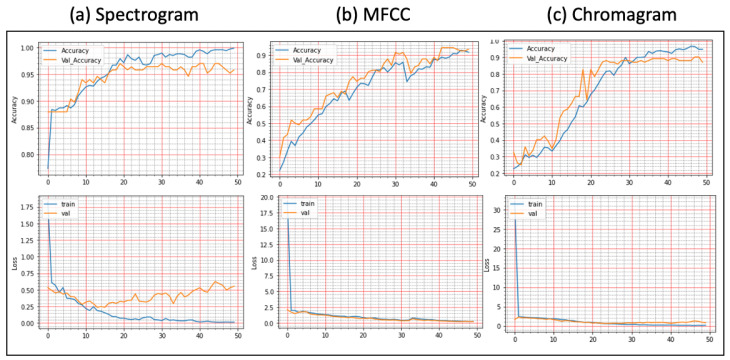
Lung Condition Classification (Accuracy vs. Loss): The Fusion Network Model (FDC-FS) was Evaluated with Three Features: (**a**) Spectrogram, (**b**) MFCC, (**c**) Chromagram.

**Results on Augmented Lung Dataset:** We achieved very impressive accuracy from the experiments with the augmented lung dataset that outperformed all state-of-the-art research. The highest accuracy was conducted by the FDC-FS model, which is the fusion of FDC-1, FDC-2, and FDC-FS. The weighted accuracy for the FDC-FS model for Spectrogram is 99.1%, the accuracy of 99% for MFCC, and 98.4% for Chromagram. Notably, it has been improved approximately 2%, 8%, 3% in Spectrogram, MFCC, and Chromagram, compared to the accuracy performance with the original data. The results for the augmented lung dataset are shown in Table 9 and Figure 7. The learning and validation graphs for learning and loss are shown in Figure 8.

**Figure 9:**
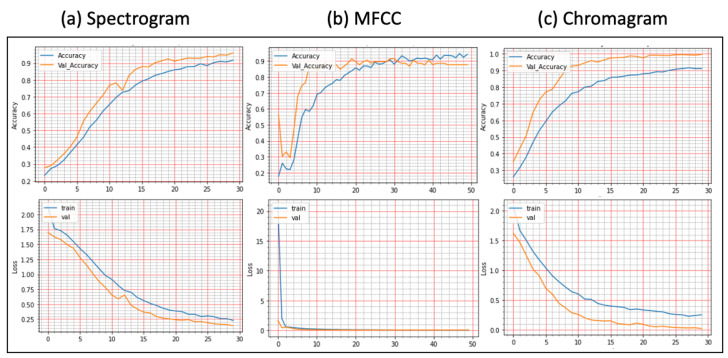
Heart Condition Classification (Accuracy vs. Loss): The Fusion Network Model (FDC-FS) was Evaluated with Three Features: (**a**) Spectrogram, (**b**) MFCC, (**c**) Chromagram.

**Results on the Augmented Heart Dataset:** For the augmented heart data experiments, we observed that FDC-2 obtained the shortest training time of 42 s for MFCC, and FDC-FS took the longest time of 4 min and 11 s for Chromagram. The FDC-FS model obtained the highest accuracy of 97% with Spectrogram and Chromagram and 96% with MFCC. From the individual model evaluation, FDC-3 obtained the highest accuracy of 96% with MFCC. It is because the FDC-3 model is bigger/deeper compared to the other two models. The learning graphs of the heart dataset for the FDC-FS model are shown in Figure 9. The overall performance of the models is given in Table 9.

**Figure 10:**
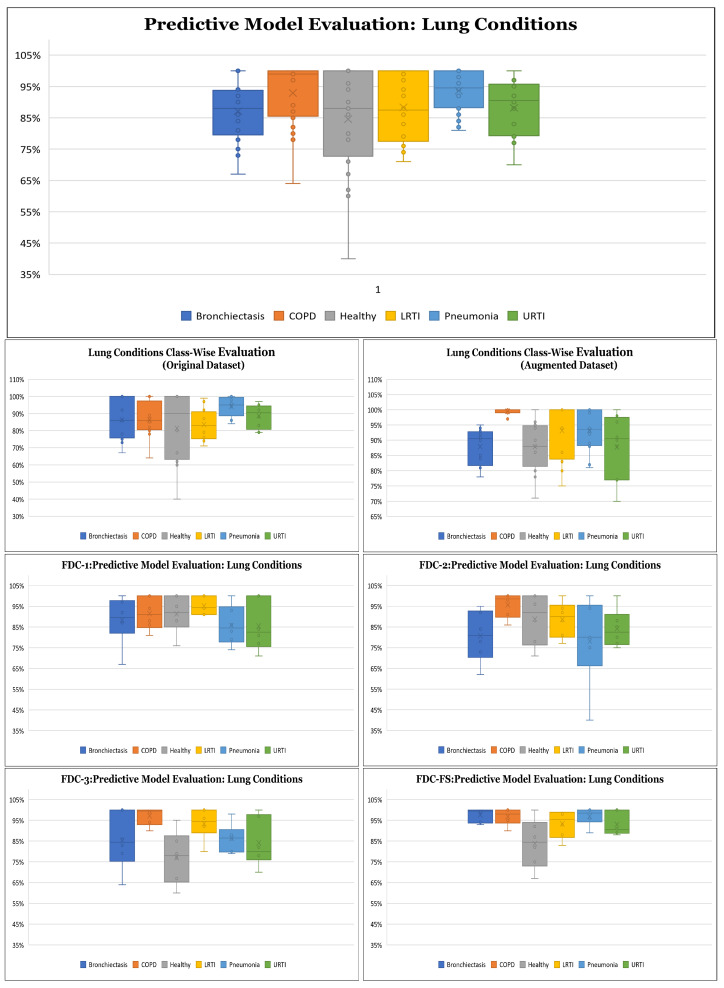
Class Wise Accuracy for Lung Condition Detection.

**Figure 11:**
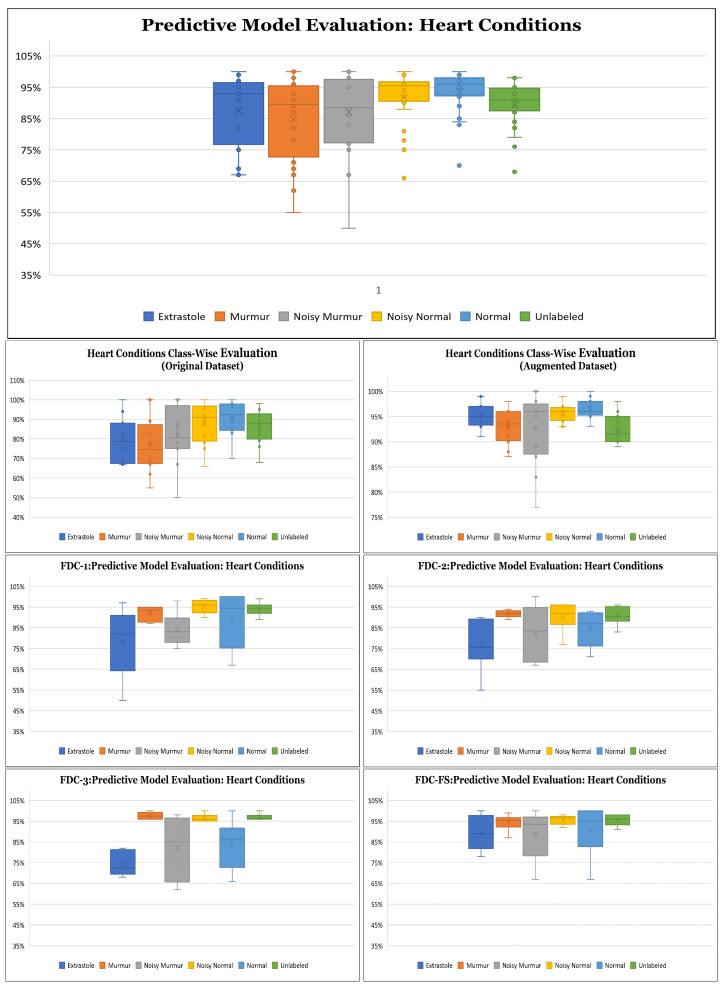
Class Wise Accuracy for Heart Condition Detection.

**Results on Class-wise Accuracy for Lung and Heart Datasets:** Class-wise accuracy for all models based on the augmented lung dataset is also shown in Figure 10 and Table 10. We obtained the highest class-wise accuracy for the COPD category based on the Spectrogram and MFCC features. For the class-wise accuracy, the highest accuracy is reported by the FDC-FS model as shown in Figure 11 and Table 11. The heart data were unbalanced but showed consistent data patterns and characteristics among categories. Thus, as the average accuracy and weighted average accuracy were similar, we constantly reported the weighted accuracy strategy. As the heart data are similar to the musical dataset, MFCC and Chromagram performed better than Spectrogram. On the other hand, Spectrogram performed very well in FDC-FS for both the heart and lung datasets. Our results are very competitive even with the state-of-the-art approaches, even with a small and reduced number of measurements.

## 5. Comparison with State-of-the-Art Research

For the comparative evaluation of our frameworks, we considered the state-of-the-art research published in reputed journals and conferences between 2018 and 2021, commonly used benchmark datasets from the ICBHI [27] and the heart challenge [57]. First, we conducted a comparative evaluation of the proposed framework (FDC) with different lung sound classification approaches [32,35,36,37,38,40]. Second, we conducted a comparative evaluation of the proposed framework (FDC) with different heart sound classification approaches [41,42,43,45,46,58,59,60].

### 5.1. Lung Sound Classification

A comprehensive evaluation of the lung sound classification models has been conducted regarding feature selection and representation, network architecture design, accuracy, and the number of trainable parameters on the lung and heart sound datasets. The best state-of-the-art approach for lung classification that has obtained the highest accuracy of 98.20% is by [60]. However, they have mixed the ICHBI dataset with their own recorded sounds from a local hospital, and another factor is that they are using shallow learning models. Ref. [38] proposed methodology obtained 98.70% accuracy, their number of trainable parameters was 3.8 M. To train their model, they needed more computational power and time. Similarly, ref. [35] used different features using the ResNet-50 model, which is a massive model with over 23 M trainable parameters. It can be seen from Table 12 that our model has shallow trainable parameters, i.e., 1.48 M, which is the lowest as compared to all state-of-the-art research. Thus, it requires very minimum resources (we mainly used CoLab for training and testing) and low epochs of only 50 for training our models. The accuracy performance is also slightly better than other approaches. FDC model also achieved the highest accuracy of approximately 99.1%. The overall comparison of our model performance for the lung sound classification with state-of-the-art research is shown in Table 12.

### 5.2. Heart Sound Classification

Similarly, we conducted a comprehensive evaluation of the heart sound classification models. Based on the number of parameters for FDC-1, the total trainable parameters are 0.24 M, and we obtained an accuracy of 93%. In contrast, for FDC-3, we received an accuracy of 96%, and the total parameters are 0.36 M. After applying the fusion technique, the parameters increased to 1.48 M with an accuracy of 97%. However, our accuracy for the fused model is near that of the state-of-the-art (approximately 98%). Still, some works did not report the trainable parameters of their proposed models [46]. Furthermore, they used the dataset with additional samples equally balanced. Therefore, it can be assumed that their trainable parameters may slightly be higher due to their network architecture of paralleling recurrent convolutional neural network (CNN), i.e., input shape, the number of layers, max-pooling, strides, the output classification size, etc. However, our accuracy is comparable to Shuvo et al. [38], whose accuracy is 97% with a model with 0.32 M trainable parameters. The overall comparison of our model performance for the heart sound classification with state-of-the-art research is shown in Table 13.

### 5.3. Discussion

Our proposed framework demonstrated superior performance compared to the state-of-the-art research both in lung and heart sound classification. We summarize the primary reasons had such good performance in lung or heart condition detection and why it consistently achieves such high performance. (i) Selection of compelling audio features to maximize the characteristics of lung or heart sounds. (ii) Application of data augmentation techniques effectively to overcome the audio data issues such as the low quality and unbalanced datasets. (iii) Transformation of the selected audio features (Spectrogram, MFCC, and Chromagram) to visual feature vectors to maximize the learning performance from deep learning. (iv) Design three unique deep neural network models (FDC-1, FDC-2, FDC-3) to discover new image patterns of audio features involved in a specific disease in lung or heart domains. (v) The fusion model (FDC-FS) is based on the transfer learning from the three different models (FDC-1, FDC-2, FDC-3) from three unique features (Spectrogram, MFCC, and Chromagram) in the lung or heart sound domains.

The limitations of the proposed framework are (i) The proposed framework performs well in two domain lung and heart sound domains; however, there is a lack of generalization. Nevertheless, we will investigate well enough to offer scientific evidence to explain why some models or specific features are better than others. (ii) We will develop suitable pattern mining methods and practices for automatic network design according to the given datasets. (iii) We will incorporate more effective transfer learning or subsequent knowledge distillation through the fusion networks that might be further optimized for the excellent balance between conciseness (fusion) and detail (specific features). (iv) We will improve pre-processing or data augmentation methods to help to overcome the data issues (noise and data imbalance), which are common in medical research, resulting in poor performance and sometimes bias in network design and parameter estimates. (v) We maintained the size of the floating window constant to maximize model efficiency. However, we can take into account the size of the floating window and then compare the output of each model.

Time is critical for our inquiry, as time-frequency images may vary between subjects due to subject-specific features and heart rate variability. As previously stated, a spectrogram is a graphical representation of a time-frequency domain signal. The signal’s frequency and sequence, as well as its temporal variations, were determined using Short Time Fourier transform (STFT). Using STFT, we were able to construct characteristics that could be used to discriminate across conditions but were unaffected by subject variance. Rather of concentrating exclusively on individual differences, we sought to discover broad characteristics that may help in correct categorization. Temporal elements are decreased when the medium changes from audio to video. By and large, our technique achieved exceptional results for a variety of reasons, including the successful extraction of audio features, their transfer to visuals, and the novel design of a deep neural network for the visual representation of heart and lung disorders.

## 6. Conclusions

In this paper, we developed the feature-based fusion network FDC-FS for the heart and lung disease classification. We used the two publicly available sound datasets with different numbers of samples and class imbalance ratios for this study. In addition, we performed our experimentation with the original dataset to compare our results with the current state-of-the-art research. The experimental results confirmed the superiority of FDC-FS that is a fusion network by combines the three unique models, i.e., FDC-1, FDC-2, and FDC-3, built with the images of specific audio features of Spectrogram, MFCC, and Chromagram. The accuracy reported for the lung dataset is 97% for Spectrogram, 91% for MFCC, and 95% for Chromagram. In contrast, for the heart data, the accuracy reported is 89% for Spectrogram, 93% for MFCC, and 92% for Chromagram.

We further improved the results by applying the data augmentation techniques to the audio clips rather than the images. We used three types of audio augmentation techniques, i.e., noise, pitch shifting, and time stretching, carefully selecting the ranges of values. As a result, the accuracy reported for the augmented lung dataset is 99.1% for Spectrogram, 99% for MFCC, and 98.4% for Chromagram. For the heart dataset, the reported accuracy is based on the accuracy of the dataset% augmentation is 97% for Spectrogram, 96% for MFCC, and 97% for Chromagram. We will further apply the proposed models and techniques to more various datasets. Moreover, we will take our research toward the multi-tasks classification by combining lung and heart models. Finally, we will extend the work for interpretable deep learning and explainable AI by providing evidence of unique patterns discovered for specific conditions.

## Figures and Tables

**Table 1:** FDC Model Hyper-parameters.

Model.	Batch Size	# of Layers	# of Hidden Layers	# of Epochs	Dropout
**Original Data**					
FDC-1	64	3	2	50	0.5
FDC-2	64	2	2	50	0.5
FDC-3	64	8	1	50	0.5
FDC-FS	64	13	3	50	0.5
**Augmented Data**					
FDC-1	128	3	2	30	0.5
FDC-2	128	2	2	30	0.5
FDC-3	128	8	1	30	0.5
FDC-FS	128	13	3	30	0.5

**Table 2:** FDC-1 Model Hyper-parameters.

Layer (Type)	Output Shape	Param #
Conv2D	(None, 124, 124, 24)	624
MaxPooling2D	(None, 31, 62, 24)	0
Activation	(None, 31, 62, 24)	0
Conv2D	(None, 27, 58, 48)	28,848
MaxPooling2D	(None, 6, 29, 48)	0
Activation	(None, 6, 29, 48)	0
Conv2D	(None, 2, 25, 48)	57,648
Activation	(None, 2, 25, 48)	0
Flatten	(None, 2400)	0
DropOut	(None, 2400)	0
Dense	(None, 64)	153,664
Activation	(None, 64)	0
DropOut	(None, 64)	0
Dense	(None, 6)	650
Activation	(None, 6)	0
Total Parameters: 241,174		
Trainable Parameters: 241,174		
Non-Trainable Parameters: 0		

**Table 3:** FDC-2 Model Hyper parameters.

Layer (Type)	Output Shape	Param #
Conv2D	(None, 126, 126, 32)	320
Activation	(None, 126, 126, 32)	0
MaxPooling2D	(None, 31, 63, 32)	0
Conv2D	(None, 29, 61, 64)	18,496
Activation	(None, 29, 61, 64)	0
MaxPooling2D	(None, 7, 30, 64)	0
Flatten	(None, 13,440)	0
Dropout	(None, 13,440)	0
Dense	(None, 64)	860,224
Activation	(None, 64)	0
Dropout	(None, 64)	0
Dense	(None, 6)	650
Activation	(None, 6)	0
Total Parameters: 879,430		
Trainable Parameters: 879,430		
Non-Trainable Parameters: 0		

**Table 4:** FDC-3 Model Hyper-Parameters.

Layer (Type)	Output Shape	Param #
Conv2D	(None, 128, 128, 32)	320
BatchNormalization	(None, 128, 128, 32)	128
Conv2D	(None, 126, 126, 32)	9248
BatchNormalization	(None, 126, 126, 32)	128
MaxPooling2D	(None, 63, 63, 32)	0
Dropout	(None, 63, 63, 32)	0
Conv2D	(None, 63, 63, 32)	18,496
BatchNormalization	(None, 63, 63, 32)	256
Conv2D	(None, 61, 61, 64)	36,928
BatchNormalization	(None, 61, 61, 64)	256
MaxPooling2D	(None, 31, 31, 64)	0
Dropout	(None, 31, 31, 64)	0
Conv2D	(None, 31, 31, 64)	36,928
BatchNormalization	(None, 31, 31, 64)	256
Conv2D	(None, 29, 29, 64)	36,928
BatchNormalization	(None, 29, 29, 64)	256
MaxPooling2D	(None, 15, 15, 64)	0
Dropout	(None, 15, 15, 64)	0
Conv2D	(None, 15, 15, 64)	73,856
BatchNormalization	(None, 15, 15, 64)	512
Conv2D	(None, 13, 13, 128)	147,584
BatchNormalization	(None, 13, 13, 128)	512
MaxPooling2D	(None, 7, 7, 128)	0
Dropout	(None, 7, 7, 128)	0
GlobalAveragePooling	(None, 128)	0
Dense	(None, 6)	1290
Activation	(None, 6)	0
Total Parameters: 363,366		
Trainable Parameters: 362,214		
Non-Trainable Parameters: 1152		

**Table 5:** FDC-FS Model Hyper-Parameters.

Layer (Type)	Output Shape	Param #
Input	(None, 128, 128, 1)	0
Conv2D	(None, 128, 128, 32)	320
BatchNormalization	(None, 128, 128, 32)	128
Conv2D	(None, 126, 126, 32)	9248
BatchNormalization	(None, 126, 126, 32)	128
MaxPooling2D	(None, 63, 63, 32)	0
DropOut	(None, 63, 63, 32)	0
Conv2D	(None, 63, 63, 64)	18,496
BatchNormalization	(None, 63, 63, 64)	256
Conv2D	(None, 61, 61, 64)	36,928
BatchNormalization	(None, 61, 61, 64)	256
MaxPooling2D	(None, 31, 31, 64)	0
DropOut	(None, 31, 31, 64)	0
Conv2D	(None, 124, 124, 24)	624
Conv2D	(None, 31, 31, 64)	36,928
MaxPooling2D	(None, 31, 62, 24)	0
BatchNormalization	(None, 31, 31, 64)	256
Activation	(None, 31, 62, 24)	0
Conv2D	(None, 126, 126, 32)	320
Conv2D	(None, 29, 29, 64)	36,928
Conv2D	(None, 27, 58, 48)	28,848
Activation	(None, 126, 126, 32)	0
BatchNormalization	(None, 29, 29, 64)	256
MaxPooling2D	(None, 6, 29, 48)	0
MaxPooling2D	(None, 31, 63, 32)	0
MaxPooling2D	(None, 15, 15, 64)	0
Activation	(None, 6, 29, 48)	0
Conv2D	(None, 29, 61, 64)	18,496
DropOut	(None, 15, 15, 64)	0
Conv2D	(None, 2, 25, 48)	57,648
Activation	(None, 29, 61, 64)	0
Conv2D	(None, 15, 15, 128)	73,856
Activation	(None, 2, 25, 48)	0
MaxPooling2D	(None, 7, 30, 64)	0
BatchNormalization	(None, 15, 15, 128)	512
Flatten	(None, 2400)	0
Flatten	(None, 13,440)	0
Conv2D	(None, 13, 13, 128)	147,584
DropOut	(None, 2400)	0
DropOut	(None, 13,440)	0
BatchNormalization	(None, 13, 13, 128)	512
Dense	(None, 64)	153,664
Dense	(None, 64)	860,224
MaxPooling2D	(None, 7,7, 128)	0
Activation	(None, 64)	0
Activation	(None, 64)	0
DropOut	(None, 7, 7, 128)	0
DropOut	(None, 64)	0
DropOut	(None, 64)	0
GlobalAveragePoolinh2D	(None, 128)	0
Concatenate	(None, 256)	0
Dense	(None, 6)	1542
Total Parameters: 1,483,958		
Trainable Parameters: 1,482,806		
Non-Trainable Parameters: 1152		

**Table 6:** Heart Sound Dataset: Original and Augmented Data.

ID	Category	Ori. Data	Aug. Data
1	Extra Systole	46	506
2	Normal	200	2200
3	Noisy Normal	120	1320
4	Murmur	66	726
5	Noisy Murmur	29	319
6	Unlabelled Test	195	2145
	Total	**656**	**7216**

**Table 7:** Lung Sound Dataset: Original and Augmented Data.

ID	Category	Ori. Data	Aug. Data
1	Bronchiectasis	29	319
2	COPD	785	8635
3	Health	35	385
4	LRTI	2	22
5	Pneumonia	37	407
6	URTI	31	341
	Total	**919**	**10,109**

**Table 8:** Lung & Heart Condition Detection with Original Data (Testing Average Accuracy).

	Lung Sound Classification				Heart Sound Classification			
**Features**	**FDC-1**	**FDC-2**	**FDC-3**	**FDC-FS**	**FDC-1**	**FDC-2**	**FDC-3**	**FDC-FS**
Spectrogram	91%	87%	84%	97%	85%	81%	73.5%	89%
MFCC	90%	93%	83%	91%	89%	91%	89%	93%
Chromagram	86%	83%	89%	95%	89%	89%	84%	92%

**Table 9:** Lung & Heart Condition Detection with Original Data (Testing Average Accuracy).

	Lung Sound Classification				Heart Sound Classification			
**Features**	**FDC-1**	**FDC-2**	**FDC-3**	**FDC-FS**	**FDC-1**	**FDC-2**	**FDC-3**	**FDC-FS**
Spectrogram	99%	98.6%	98.3%	99.1%	93%	92%	95.5%	97%
MFCC	99.3%	98%	98.2%	99%	95%	93%	96%	96%
Chromagram	97%	97%	95%	98.4%	94%	93%	95%	97%

**Table 10:** Class Wise Accuracy for Lung Condition Detection.

Model	Feature	Class	Bronchiectasis	COPD	Healthy	LRTI	Pneumonia	URTI	W. AVG
FDC-1	Spec.	Ori.	92%	100%	67%	87%	87%	97%	91%
		Aug.	81%	100%	94%	86%	100%	88%	99%
	MFCC	Ori.	100%	89%	100%	76%	95%	88%	90%
		Aug.	91%	100%	95%	100%	94%	91%	99.3%
	Chroma	Ori.	79%	83%	100%	74%	86%	93%	86%
		Aug.	84%	100%	71%	100%	81%	77%	97%
FDC-2	Spec.	Ori.	73%	78%	62%	92%	84%	95%	87%
		Aug.	91%	99%	86%	100%	100%	98%	98.6%
	MFCC	Ori.	78%	88%	100%	71%	100%	96%	93%
		Aug.	81%	100%	88%	94%	92%	77%	98%
	Chroma	Ori.	100%	80%	40%	75%	94%	80%	83%
		Aug.	85%	100%	80%	75%	88%	77%	97%
FDC-3	Spec.	Ori.	86%	64%	100%	79%	100%	83%	84%
		Aug.	94%	99%	100%	100%	100%	90%	98.3%
	MFCC	Ori.	67%	85%	60%	77%	95%	79%	83%
		Aug.	69%	100%	59%	50%	77%	74%	98.2%
	Chroma	Ori.	86%	87%	80%	88%	98%	79%	89%
		Aug.	78%	97%	78%	100%	82%	70%	95%
FDC-FS	Spec.	Ori.	100%	100%	100%	99%	94%	93%	97%
		Aug.	95%	100%	90%	100%	100%	96%	99.1%
	MFCC	Ori.	75%	82%	67%	87%	100%	92%	91%
		Aug.	92%	100%	96%	80%	93%	100%	99%
	Chroma	Ori.	100%	100%	100%	97%	96%	89%	95%
		Aug.	90%	100%	88%	100%	89%	91%	98.4%

**Table 11:** Class Wise Accuracy for Heart Condition Detection.

Model	Feature	Class	Extrastole	Murmur	Noisy Murmur	Noisy Normal	Normal	Unlabeled	W. AVG
FDC-1	Spec.	Ori.	69%	89%	50%	97%	85%	79%	85%
		Aug.	94%	88%	87%	93%	95%	95%	93%
	MFCC	Ori.	83%	79%	83%	75%	98%	87%	89%
		Aug.	99%	93%	96%	96%	98%	90%	95%
	Chroma	Ori.	100%	67%	78%	100%	97%	92%	89%
		Aug.	95%	94%	95%	93%	99%	89%	94%
FDC-2	Spec.	Ori.	75%	55%	75%	90%	89%	76%	81%
		Aug.	93%	91%	89%	94%	93%	91%	92%
	MFCC	Ori.	75%	69%	67%	92%	100%	93%	91%
		Aug.	93%	91%	77%	96%	96%	90%	93%
	Chroma	Ori.	88%	71%	86%	78%	93%	92%	89%
		Aug.	91%	90%	83%	96%	95%	90%	93%
FDC-3	Spec.	Ori.	82%	70%	75%	81%	70%	68%	73%
		Aug.	97%	96%	100%	99%	96%	98%	92%
	MFCC	Ori.	67%	62%	88%	96%	98%	82%	89%
		Aug.	95%	96%	100%	96%	97%	95%	96%
	Chroma	Ori.	88%	100%	75%	66%	84%	89%	84%
		Aug.	97%	96%	96%	97%	100%	96%	95%
FDC-FS	Spec.	Ori.	94%	78%	100%	97%	83%	84%	89%
		Aug.	99%	87%	96%	95%	96%	94%	97%
	MFCC	Ori.	67%	82%	100%	92%	96%	95%	93%
		Aug.	97%	94%	96%	97%	98%	92%	96%
	Chroma	Ori.	67%	100%	100%	88%	92%	98%	92%
		Aug.	94%	98%	98%	96%	96%	91%	97%

**Table 12:** State-of-the-art Lung Classification Models.

Work	Method	Network	Class#	Data	Para#	Aug	Feature	Results
Dalal (2018) [32]	Ensemble	SVM, KNN, GMM, CNN	7	R.A.L.E data	NA	✓	MFCC, LBP	ACC: 95.56%
Hai (2019) [35]	NA	ResNet50	3	489	+23 M		Sp	ACC: 98.79%
Fatih (2020) [36]	Ensemble	CNN	4	920	NA		Sp Images, Deep Features	ACC: 71.15%
Demir (2020) [37]	NA	CNN, SVM	4	6898	138 M		Sp	ACC: 65.9%
Demir (2020) [37]	Transfer learning	CNN, SVM	4	6898	138 M		Sp Images	ACC: 63.09%
Samiul (2020) [38]	NA	CNN	6	917	3.8 M	✓	Hybrid Scalogram	98.70%
Luay (2021) [40]	Ensemble	SVM, KNN, DT, KNN	6	308/1176	NA		Entropy features	ACC: 98.20%
FDC-FS (Ours)	Fusion	DCNN	6	Original (Augmented): 919 (10,109)	**1.48 M**	✓	SP, MFCC, CH	**ACC: 99.1%**

**Table 13:** State-of-the-art Heart Classification Models.

Work	Method	Network	Class#	Data	Para#	Aug	Features	Results
Potes (2016) [41]	Ensemble	CNN	2	Normal (Abnormal): 2575 (665)	NA		MFCC	ACC: 85%
Zhang (2017) [42]	NA	CNN+SVM	3/4	Heart sounds 1 & 2	NA		SP	Precision: 77%/71%
Bozkurt (2018) [43]	NA	CNN	4	PhysioNet: Abnormal (Normal)	NA	✓	MFCC, Mel-SP	ACC: 81.50%
Wu (2019) [58]	Ensemble	CNN	2	Normal (Abnormal): 2575 (665)	61 M		Sp, Mel-SP, MFCC	ACC: 86%
Shu (2020) [45]	NA	WaveNet	5	1000	0.32 M		Multiple features	Training ACC: 97%
Xiao (2020) [59]	Transition	1D CNN	4	PhysioNet: 3153	0.19M		Raw signal w/t band filter	ACC: 93%
Muqing (2020) [46]	Concat.	CRNN, PRCNN	4	PhysioNet: 3240	NA		MFCC	ACC: 98%
Koike (2020) [61]	Pre-train.	PANN	2	PhysioNet:	80.7M	NA	Log-Mel	UAR: 89.7%
Mehmat (2021) [60]	NA	1D CNN	4	PhysioNet	NA		LBP+LTP	ACC: 91%
FDC-FS (Ours)	Fusion	DCNN	6	Original (Augmented): 656 (7216)	**1.48 M**	✓	SP, MFCC, CH	**ACC: 97%**

## Data Availability

Not applicable.
